# Oxidative-Stress-Induced Cellular Toxicity and Glycoxidation of Biomolecules by Cosmetic Products under Sunlight Exposure

**DOI:** 10.3390/antiox10071008

**Published:** 2021-06-23

**Authors:** Syed Faiz Mujtaba, Agha Parvez Masih, Ibrahim Alqasmi, Ahmad Alsulimani, Faizan Haider Khan, Shafiul Haque

**Affiliations:** 1 Department of Zoology, Faculty of Science, Shia Post Graduate College, Sitapur Road, Lucknow 226020, India; faizmujtaba42@gmail.com (S.F.M.); aghapervez@gmail.com (A.P.M.); 2 Public Health Department, Saudi Electronic University-Jeddah Branch, Jeddah 23442, Saudi Arabia; i.alqasmi@seu.edu.sa; 3 Medical Laboratory Technology Department, College of Applied Medical Sciences, Jazan University, Jazan 45142, Saudi Arabia; ahmad.alsulimani@hotmail.com; 4 Department of Pathology, Lambe Institute for Translational Research, School of Medicine, National University of Ireland Galway (NUIG), H91V4AY Galway, Ireland; faizan.khan@nuigalway.ie; 5 Research and Scientific Studies Unit, College of Nursing and Allied Health Sciences, Jazan University, Jazan 45142, Saudi Arabia

**Keywords:** cosmetics, photosensitization, photoproducts, UV-R

## Abstract

Cosmetics, commonly known as ‘makeup’ are products that can enhance the appearance of the human body. Cosmetic products include hair dyes, shampoos, skincare, sunscreens, kajal, and other makeup products. Cosmetics are generally applied throughout the face and over the neck region. Sunlight has different wavelengths of light, which include UV-A, UV-B, UV-C, and other radiations. Most cosmetic products have absorption maxima (λmax) in the range of visible light and UV-R. The effect of light-induced photosensitization of cosmetic products, which results in the production of free radicals through type-I and type-II photosensitization mechanisms. Free-radicals-mediated DNA damage and oxidative stress are common consequences of cosmetic phototoxicity. Cosmetic phototoxicity may include percutaneous absorption, skin irritation, eye irritation, photosensitization, mutagenicity, and genotoxicity. Oxidative stress induces membrane lipid peroxidation, glycoxidation, and protein covalent modifications, resulting in their dysfunction. Natural antioxidants inhibit oxidative-stress-induced cosmetic toxicity. Sunlight-induced photodegradation and accumulation of cosmetic photoproducts are also a matter of serious concern. India has tropical weather conditions throughout the year and generally, a majority of human activities such as commerce, agriculture, sports, etc. are performed under bright sunlight conditions. Thus, more focused and dedicated research is warranted to explore the effects of cosmetics on oxidative stress, glycoxidation of biomolecules, and photoproducts accumulation for its total human safety.

## 1. Introduction

Cosmetics (also referred to as personal care products) are products usually made to cleanse, enhance beauty, prevent skin tanning, increase attractiveness, enrich facial expressions, or altering the appearance without compromising the body’s functions or structure. The chemical constituents present in cosmetics such as sunscreen, lipsticks, and hair dyes can cause adverse effects in the form of skin allergy, contact dermatitis, or other skin-related diseases [1]. The problems related to cosmetics’ toxicity may be short term and go away if the use of the product is stopped. A majority of the cosmetic products contain water and nutrients that provide utopia for microbial growth and survival. Skin infections can be caused by contaminated hair dyes, body lotions, and sunscreens, particularly when they are applied to dried, cracked, or compromised skin. Earlier studies have suggested that the use of contaminated mascara can even cause blindness [2]. It has been reported that cosmetic products such as hair dye, shampoo, etc. contain ingredients that are probably human carcinogens. Those ingredients have been proven to be “penetration enhancers,” which can increase absorption through the skin. No major research has been carried out so far regarding cosmetics’ safety and its associated health risks for the chemical ingredients present in them. However, the US Food and Drug Administration and European Union have introduced guidelines to help consumers to select products based on their sun protection factor (SPF) and protection against ultraviolet (UV) radiation [3]. A list of daily use personal care cosmetic products’ ingredients and their related toxic effects are provided in Table 1. Studies have proven that an average adult uses nine cosmetic products daily. Dogra et al. (2003) reported that the incidence of contact dermatitis is around 3.3% among users of various ingredients of cosmetics. Contact allergic dermatitis, the most common type of adverse effect through cosmetic products, has an incidence of 59.2%, caused mainly due to shaving creams, hair dyes, and lipsticks [4]. Cosmetic products can prove to be benign, but they can cause cancer when used for a longer duration. Other cosmetics-associated problems are mutations, allergic reactions, respiratory problems, and several other physiological and reproductive problems. The Center for Disease Control (CDC) has reported that a majority of the people using cosmetics are exposed to phthalates, a family of chemicals generally used in cosmetic products [5]. Parabens has been shown to have an effect on sperm production at low doses in male juvenile rats [6]. Hans et al. (2008) studied the phototoxicity of cosmetic products, which can produce reactive oxygen species (ROS), cause hemolysis, and participate in peroxidation of lipid moieties in human red blood corpuscles (RBCs) (in vitro) under sunlight exposure; therefore, sunlight exposure must be avoided following the application of cosmetics, and this is why it is suggested not to go out in bright sunlight after the application of photosensitive cosmetic products [7]. 

Hair dyes, which are categorized under cosmetic products may cause major adverse effects [8]. More than 5000 different chemicals are being identified in hair dyes, and many of them have been proven to be carcinogenic to animals [9,10]. As hair dyes are frequently being applied by a major population of the world, it is of utmost importance to study the associated risks and potential of causing cancer. Today, the use of dyes has been increased significantly, and the International Agency for Research of Cancer (IARC) stated that women (35%) are more prone to cancer in comparison to men (10%) due to their high usage of hair dyes [11].

There are many studies that reported the dermal absorption of sunscreen creams/lotions and their delivery or presence in blood and urine. Earlier studies showed that exposure of UV light to α-hydroxy acids can cause DNA damage and the formation of sunburn cells, and these α-hydroxy acids are extensively used in cosmetics as an antiaging compound [12]. Benzophenone, a well-known ingredient of sunscreens, has been discovered in human breast milk [13]. Various studies have proved the toxic effects of sunscreen ingredients on the environment. Many anthropogenic activities are also responsible for introducing sunscreen products to the environment such as bathing, swimming, etc [14]. Many cosmetics that are applied to the eyes, such as eye shadow, show adverse effects on eyelids. Kajal and Mascara, an eye cosmetic can cause harmful impacts, particularly *Pseudomonas aeroginosa* corneal infections; it can also contain mercury and lead, which results in adverse health issues [15].

Alterations in the stratospheric ozone as well as changes in the climate over the last 40 years have distorted the solar ultraviolet (UV) radiation conditions on the Earth’s surface [16]. The UV radiation reaching the Earth’s surface comprises UV-A (~95%) and a small fraction of UV-B (~5%). Due to the popularity of tanning salons and changes in lifestyle, humans are continually being exposed to UV-A [17]. UV radiation from sunlight is the major culprit of various biological effects, including structural changes in DNA, proteins, and many other biologically active molecules, mutations, chronic depression of important physiological processes, acute physiological stress resulting in growth reduction and cell division, and photocarcinogenesis [18,19]. Excitation and compound modification of cosmetic ingredients through UV exposure increases its toxic effect. Cosmetic products are capable of absorbing UV and visible light. After absorbing the light, cosmetic ingredients are excited to higher energy levels that can initiate various excited-state reactions, leading to the generation of oxidative stress, DNA damage, protein, and cell membrane [20]. Previous data showed the toxic effects of cosmetic ingredients under sunlight exposure. Cosmetic ingredients show their adverse effects on the skin, but they can also reach other organs and show their toxicity via blood; they can also enter the environment through the skin during swimming and bathing, as well as urination [21]. Thus, this review focuses on the adverse effects of the application of cosmetics on human skin and its adverse consequences in the form of DNA damage, phototoxicity, and glycoxidation of biomolecules through the generation of oxidative stress. Thus, it is essential to make people sensitized regarding the usage of cosmetics, in addition to avoiding outdoor activities during peak hours of sunlight exposure for human safety.

## 2. Sunlight/UV-R-Induced Photosensitization of Cosmetics 

**Figure 1:**
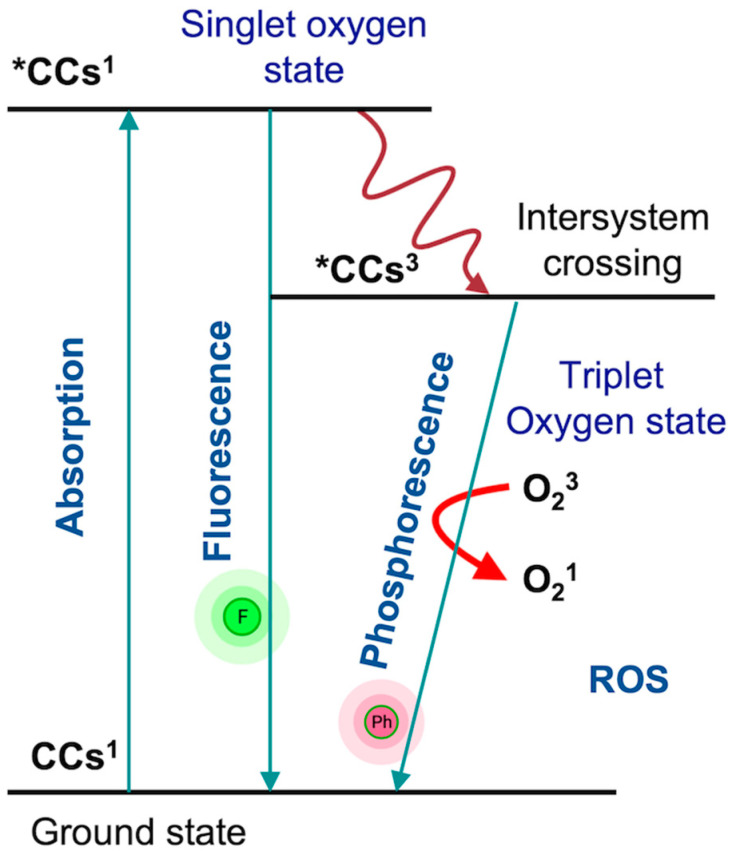
Diagrammatic representation of the electronic energy of the physical events supplementing the absorption of photon by cosmetic chemicals (CCs): CCs^1^, ground state; *CCs^1^, excited singlet state; *CCs^3^, excited triplet state; O_2_^3^, triplet (ground) state oxygen; O_2_^1^, singlet (excited) state oxygen.

Many cosmetic ingredients show absorption maxima (λmax) under UV-A, UV-B, as well as the visible wavelength of sunlight. UV-R-induced photosensitization of cosmetic products leads to two types of reactions, i.e., type I and type II reaction: type I photosensitization occurs when compound reacts to the excited state of the sensitizer, which leads to the formation of radicals or radical ions through electron transfer or hydrogen atom, but in the case of type II photoreaction, the excited sensitizer reacts with oxygen for forming a singlet molecular oxygen. UV-R-induced photosensitized cosmetic products lead to the generation of reactive oxygen species (ROS), which is responsible for the oxidation of biological molecules, cell membranes, etc. In general, different cosmetic products have different levels of toxicity under sunlight exposure. Cosmetic ingredients such as hair dye, lipstick, mascara, sunscreen, etc. all show different levels of phototoxicity due to their different absorption spectra under sunlight and generation of oxidative-stress-mediated toxicity. Photosensitized products lead to the formation of various free radicals such as ^1^O_2_, O_2_^•^ and ^•^OH radicals, which can cause oxidative-stress-mediated cellular toxicity [22] (Figure 1). 

## 3. Photocytotoxicity of Hair Dyes

Hair dyes are equally popular among elderly and young people, both using them to color their hair for fashion as well as for concealing gray hair (or in the case of concealing premature gray hair). People working in hair dye industries and those who intentionally apply hair dyes inevitably are exposed to sunlight and have various skin problems. The photosensitization of hair dye and its effect on human skin is well documented. Previous studies have proved that hair dyes can penetrate the skin [23]. When applied to the skin, the combination of light irradiation and contamination with hair dye ingredients may cause adverse effects on human health [24]. Hair dyes have been proven to be photosensitive for a long period of time [24]. According to the European Commission Scientific Committee Safety, 46 hair dye substances act as sensitizers; among them, 10 act as extreme (including p-phenylenediamine), 13 as strong, and 4 act as moderate sensitizers [25]. The contact-related allergic reactions caused due to hair dye ingredients may vary from minor contact dermatitis to fatal life-threatening problems such as bronchospasm, angioedema, asthma, etc [26,27]. Various allergic reactions showing diverse adverse effects due to the application of phenylenediamine and other hair dye ingredients have been increased in recent times [25]; moreover, various allergic reactions including coma and death have been linked with the use of hair dyes. Toxic effects of hair dye reactions cannot be diagnosed by the person using it unless it is visibly apparent on the skin of the applicant. The incidence or quantum of dermatitis caused by hair dye use is much more than reported in the literature [28]. The main symptoms of hair-dye-induced toxicity may include severe edema of the face, scalp, and ears. Ingredients in hair dyes are absorbed through inhalation and skin contact, resulting in damage to the skin and respiratory tract, even cancer [29,30]. O_2_^−^ and ^•^OH radicals are formed via type-I photosensitization reaction in the presence of paraphenylenediamine (PPD), which leads to DNA damage and apoptosis in the skin keratinocytes [31]. The most common clinical presentation includes contact dermatitis to the photo-exposed skin, erythema, lesions, etc. the National Cancer Institute, USA, reports that 2% of all cases among women with non-Hodgkin’s lymphoma are because of routine use of various hair dyes available commercially. Additionally, hair dyes may pose a serious risk to children before and after the pregnancy, even in cases, in which mothers used those products shortly prior to conception or during the pregnancy [32]. From a public health perspective, hair dye toxicity should be taken into account as an important factor to reduce the lethal effect caused by hair dyes, especially to people living in tropical areas where sunlight exposure is high during peak hours due to their main outdoor activities.

## 4. Adverse Effects of Sunscreen under Sunlight Exposure

Sunscreens are types of compounds that are capable of preventing ultraviolet (UV) radiation to penetrate the skin because of the UV filter present in them. Cosmetic ingredients are used to protect against harmful radiations from the sunlight. Although it is assumed that sunscreens are safe against the harmful radiation of sunlight, previous studies have reported that ingredients in sunlight may absorb UV-R and generate oxidative stress [20]. Sunscreen ingredients have been proven to be one of the potent endocrine disruptors. As of now, other than sunscreens, no other options are available for protecting the skin from sunlight’s harmful UV radiation. Frequent usage of sunscreens across the world in recent years has increased the frequency of sun-related pathologies, especially a significant rise in the cases of malignant melanoma. Animal and human studies have proved that most of the sunscreen ingredients have neurotoxic potential [33]. Sunscreen ingredients may also cause reproductive/development toxicity [14]. Most of the sunscreen ingredients show absorption under UV-A, UV-B, and visible spectra of sunlight [34]. Benzophenone induces oxidative stress under sunlight exposure through the generation of (^1^O_2_, O_2_^−^ and ^•^OH) [20]. Hence, some safer alternatives should be preferred for skin protection against the harmful radiation of sunlight.

## 5. Lipstick, Kajal, and Surma as a Health Hazard

Lipstick is a cosmetic product containing pigment, oils, and waxes that provide color, texture, and protection to the lips. Previously conducted studies have proved the presence of heavy metals such as lead in cosmetic ingredients [35]. Lipstick ingredients have photosensitizing potential due to which sunlight exposure should be avoided during peak hours [7].

Kajal is very popular among females across the world for enhancing the beauty of the eyes. Due to its black color, it is being reported that it contains different amounts of PAHs [36]. In India, along with Kajal, Surma is equally popular for frequent ophthalmic use and work as a beautifying cosmetic. Earlier studies have proved the presence of heavy metals in Kajal products. Long-term use of Kajal may lead to Pb (lead) accumulation in the human body, affecting bone marrow and brain, as well as causing anemia and convulsions [37]. Kajal ingredients are not restricted to the eye alone; they are absorbed through the cornea and distributed throughout the body [38]. The well-known effects of heavy metals and PAHs in Kajal emphasize its potential health risk for its users. 

## 6. Toxic Effect of Cosmetics on the Biological System

Previous studies have reported that photosensitized cosmetics induce ROS generation and DNA damage [39]. UV-R-induced, oxidative-stress-mediated DNA damage by cosmetic ingredients may include single- and double-strand breakage, cyclobutane pyrimidine dimers (CPDs) formation, and 6-4 photoproducts [40]. Photosensitized cosmetics induce ROS formation under oxidative-stress environments, leading to the induction of lipid peroxidation and glycoxidation reactions that results in the elevated endogenous production of reactive aldehydes and their derivatives, which offer advanced lipid oxidation and glycation-end products [41]. Generation of ^1^O_2_, O_2_^−^ and ^•^OH radical are being reported in sunscreens and hair dye phototoxicity [31,42]. Cosmetic ingredients in sunscreens induce UV-mediated oxidative stress in skin cells [43]. Earlier studies have reported that some of the hair dye constituents are potential nephrotoxic agents and cause severe nephrotoxicity in humans [44]. Previous reports have documented that most cosmetic products contain heavy metals in the form of lead (Pb), mercury (Hg), etc., which can cause inflammation of the liver, kidneys, and urinary tract [45]. 

## 7. Role of Antioxidants in Cosmetic Toxicity

The human body is equipped with a variety of antioxidants that serve to counterbalance the effect of oxidants. Antioxidants include low molecular weight compounds, such as vitamins (vitamin C and vitamin E), β-carotene, uric acid, GSH, etc. Vitamin C offers intracellular as well as extracellular antioxidant capability, predominantly by scavenging oxygen free radicals, while Vitamin E is concentrated in the hydrophobic interior of the cell membrane for protecting membrane damage caused by ROS generation [46]. To protect the skin from the harmful effects of ROS, the most suitable formulation of cosmetics must supplement antioxidant molecules such as vitamin A, α-tocopherol (vitamin E), or ascorbic acid (vitamin C) for scavenging the ROS before they reach the dermis and therefore prevent molecular and cellular skin damage [47].

Vitamin E has been shown to protect the skin in the presence of sunscreens, and studies have found that it also reduces lipid peroxidation. Previous studies have reported that topical application of vitamin C inhibits the formation of sunburn cells [48]. Darr et al. (1992) reported that topically administered vitamin C can inhibit UV-mediated phototoxicity in pigskin [48]. Human skin has several natural endogenous enzymatic antioxidants, such as GSH peroxidase, catalase, and SOD, along with some nonenzymatic low-molecular-weight antioxidants, e.g., vitamin E, vitamin C, uric acid, GSH, and ubiquinol [49].

It can be stated that oxidative stress induced by cosmetic ingredients can pose a serious threat to skin cells, which can be minimized through the intake or application of endogenous and exogenous antioxidants, which could play an important role in suppressing the effect of ROS.

## 8. Discussion

**Figure 2:**
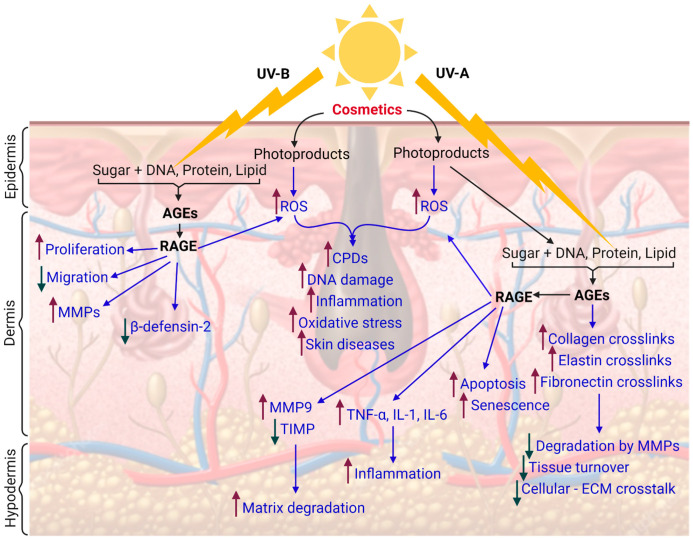
Schematic representation showing the effects of UV-induced cosmetics photosensitization, advanced glycation end (AGE) products, and receptor for AGEs (RAGE) on the human skin. UV-B can penetrate the epidermis, while UV-A can penetrate deeper in the dermis and induce the biomolecules to form AGE that may influence several targets in the skin through receptor- and nonreceptor-facilitated pathways (created with BioRender.com).

The theme of the review is to elucidate the potential health hazard posed by cosmetics (Figure 2). Apart from heavy metals such as Hg and Pb, a variety of established toxic chemicals are still being used as common ingredients in cosmetics, which are prohibited by many countries. For example, it is well established by earlier research studies that some hair dyes and cosmetics have a potential role in inducing carcinogenesis [50].

The chemical ingredients present in cosmetic products may be taken up or absorbed by the body either through inhalation or through skin contact, resulting in various skin diseases and long-term respiratory tract disorders [10,51]. Past studies have reported that cancer, renal failure, diabetes mellitus, hypertension, yellowish-brown coloration, multiple stretch marks, and skin rashes are few of the health and toxicological hazards linked with cosmetic product usage and are associated with toxic chemicals used in the preparation of cosmetics [52]. The environmental working group (EWG) has provided guidelines for safe sunscreens. EWG tested nearly 2000 sunscreens from more than 27 brands, and the researchers found that more than 75% of them had toxic chemicals capable of increasing the risk of cancer and other severe health concerns. Sunscreens mostly enter the body through dermal exposure; however, eating and drinking with hands applied with sunscreens result in oral, gastrointestinal, and pulmonary exposure of sunscreens [33]. Mostly, the products in cosmetics show absorption under UV-R and visible light [53]. Cosmetic ingredients possess chromophore groups capable of performing the absorption process of electromagnetic radiation and thereby could act as photosensitizers [54]. Following light absorption, cosmetic products generally become photosensitized, which results in photoallergic, phototoxic, irritant contact dermatitis, and anaphylactic reactions [55]. Photosensitized beauty products can induce oxidative stress through the generation of ^1^O_2_, O_2_^−^ and ^•^OH radicals [20]. Photosensitized cosmetics induce cytotoxicity and DNA damage under UV-R/sunlight exposure [31]. Photosensitized cosmetic products trigger a variety of pathological conditions that include DNA damage such as single- and double-strand breakage, micronuclei, and cyclobutane pyrimidine dimers (CPDs) formation through ROS generation within the cell, leading to carcinogenesis and mutations [56]. Application of cosmetic products and outdoor activities may have a serious impact on human health as cosmetics are not restricted to the skin alone but are absorbed through the skin, which characterizes their effects as systemic [57]. Photo-modification is another serious threat to cosmetic users as cosmetic photoproducts formed under sunlight exposure may even enhance cosmetic toxicity [20,31]. Hence, future research studies are warranted to appraise the extent to which photoproducts may occur on human skin under natural and normal sunlight exposure conditions and their phototoxicity in our day-to-day life. Previous studies showed the cytotoxicity of cosmetic products in sea urchin eggs, which results in alteration in calcium homeostasis, protein and DNA synthesis, protein phosphorylation, intracellular pH, and sodium and potassium content [58]. Para-phenylenediamine (PPD) hair dyes can be detected in urine 48 hr after its application, highlighting the potential health hazard of this dye, which can penetrate the body [59]. On the other hand, a significant number of oxidative hair dye reaction products are systemically available to the consumers. Hence, for hair dyes, detailed chemical testing is desired. According to an estimate, more than one-thirds of women above 18 years old and 10% of men above 40 years old use some sort of hair dye product [60]. As the use of oxidative hair dyeing is becoming popular all over the world and among all ages irrespective of the gender, their associated safety issues must be appraised in detail. Furthermore, oxidative stress mediated by cosmetic products may lead to lipid oxidation, glycation and protein oxidation, which have a pathogenic role in development and progression of different oxidative-based diseases including diabetes, atherosclerosis, and neurological disorders. 

Thus, before reaching the consumer, cosmetic products must be checked for their safety by authorized regulatory bodies, and only when they are proved to be safe for human use can they be allowed for use in a specified type of cosmetic formulation within a prescribed concentration limit.

## 9. Conclusions

The rising demand for cosmetic products across the globe among adults and teens has generated an understanding of safety issues associated with their use. The main objective of this review is to highlight the toxicological effects of chemical ingredients of cosmetics and personal care products and highlight their long-term use associated with possible health hazards. Cosmetic ingredients are a type of silent enemy to humans, and continued exposure may pose serious health concerns to the users. As reported in the past, many cosmetic products, predominantly the constituents in sunscreens and hair dyes, have been categorized as probable human carcinogens. Such chemical ingredients have penetration enhancers that facilitate skin penetration and toxicity. Phototoxicity of cosmetic constituents can cause long-standing local as well as systemic toxic effects, possibly by lipid peroxidation.

Overall, this review highlights the adverse health effects to the users of cosmetics, especially when exposed to bright sunlight during which they become photosensitized and induce oxidative-stress-mediated cytotoxicity; therefore, some safer alternatives should be preferred and pondered for the total safety of human beings.

## Figures and Tables

**Table 1:** List of hazardous chemicals in cosmetic products and their related health hazards.

S. No.	Chemicals	Products	Health Impacts
1.	Phthalates	Fragrances product	Damage to reproductive system and development
2.	Parabens	Shampoo, shaving cream, moisturizers, makeup	Allergic reaction, lesions, scaly red rash
3.	1,4 dioxane	Detergents, foams, stabilizers, solvents	Cancer-causing agent
4.	Asbestos	Makeup and dye products	Allergic reaction, eye sight effect
5.	Lead	Hair dye and eye makeup	Neurotoxic in nature, causes behavioral changes and decrease in learning process, reproductive toxic having carcinogenic effects
6.	Formaldehyde and Butyl Acetate	Nail paints, hair smoothing agents	Dizziness or drowsiness, skin crack, and dry skin
7.	Parabens	Deodorant, shampoo, cream, baby products, makeup, etc.	Ethyl, methyl, propyl, isobutyl, butyl, and related parabens are generally reproductive toxic
8.	Phenylenediamine (PPD)	Hair dye	Cause mutations and carcinogenic effect; skin sensitivity and respiratory disorders
9.	Talc	Baby powder, deodorant, soap	Human carcinogen, ovarian cancer
10.	Toulene	Nail polish and hair dye	Reproductive and developmental damage
